# Sleeplessness

**Published:** 1885-05

**Authors:** 


### Sleeplessness

Is the result of over bodily or mental effort. When a man works beyond his strength, or thinks or studies more than rest can restore, then, sooner or later, comes that inability to sleep soundly, that wakefulness, which is more wearing even than bodily labor, and which feeds the debility which first gave rise to it. The result is, a man is always tired, never feels rested, even when he leaves his bed in the morning; hence he wastes away, and finds repose only in the grave; if indeed, insanity do not supervene. It is too often a malady, remediless by medical means. Avoid then, as you would a viper or a murderer, all over effort of mind and body; it is suicidal. Whatever you do, get enough sleep; whatever you do, take enough rest to restore the used energies of each preceeding twenty-four hours; if you do not, you may escape for a few months, and if posessing a good constitution, years may pass away before any decided ill result forces itself on your attention; but rest assured, the time will come, when the too often baffled system, like a baffled horse, will refuse to work; it will not take prompt and sound sleep; it will not be rested by repose, and that irritating wakefulness will come upon you, which philosophy cannot conquer, which medicine cannot cure; and wasting by slow degrees to skin and bone, rest is found only in the grave.

